# Zlig ligament reconstruction versus tibial plateau leveling osteotomy for cranial cruciate ligament rupture in dogs: a prospective controlled study

**DOI:** 10.3389/fvets.2026.1936682

**Published:** 2026-09-10

**Authors:** S. Chwatal, F. Volz, M. Kornmayer, Y. Zablotski, A. Meyer-Lindenberg

**Affiliations:** 1 Clinic for Small Animal Surgery and Reproduction, Ludwig-Maximilians-University, Munich, Germany; 2 Clinic for Small Animal Surgery and Reproduction, Munich, Germany; 3 Faculty of Veterinary Medicine, Clinic for Small Animal Surgery and Reproduction, Ludwig-Maximilians-University, Munich, Germany

**Keywords:** cranial cruciate ligament disease, dog, stifle surgery, synthetic ligament reconstruction, tibial plateau leveling osteotomy (TPLO), Zlig

## Abstract

**Objectives:**

Cranial cruciate ligament rupture (CCLR) is one of the most common causes of hindlimb lameness in dogs, causing stifle instability, osteoarthritis and damage to the medial meniscus. While tibial plateau leveling osteotomy (TPLO) is the most common performed method for cranial cruciate ligament rupture in dogs, intra-articular reconstruction of the cranial cruciate ligament is the gold standard in human medicine. The objective of this prospective clinical study was to investigate whether the synthetic ligament Zlig by Eickemeyer® for intra-articular reconstruction can achieve similar clinical and gait analysis results in cases of CCLR in dogs compared to TPLO.

**Methods:**

Thirty dogs with CCLR were enrolled in this prospective clinical study at the small animal clinic at LMU Munich. The dogs underwent surgery with TPLO (*n* = 16) or the synthetic intra-articular ligament Zlig (*n* = 14) and were assessed preoperatively as well as 6, 12 and 18 weeks postoperatively. Clinical assessment included lameness scoring, joint effusion, pain response during clinical examination and tibial compression test. Objective gait analysis was performed using treadmill-based force plate and motion capture systems.

**Results:**

Both groups showed significant clinical and functional improvement over the 18-week period. At 12 weeks, the TPLO group exhibited significantly greater reductions in lameness score, joint effusion and pain sensitivity of the stifle. At the all other time points, there were no significant differences between the TPLO group and the Zlig group. By week 18, all dogs treated with TPLO had a negative tibial compression test, while 78.6% (*n* = 11) of the dogs in the Zlig group were still tested positive. Total postoperative complication rate was 18.8% (*n* = 3) in the TPLO group and 35.7% (*n* = 5) in the Zlig group.

**Conclusion:**

Both surgical techniques resulted in improved limb function. The TPLO group showed more reliable stabilization and a lower complication rate in comparison to the Zlig group. The role of the residual instability in the dogs of the Zlig group needs further studies examining its biomechanical properties. Whether the intra-articular Zlig implant represents a potential alternative for the future needs to be further validated through more prospective studies over a longer time period.

## 1 Introduction

Rupture of the cranial cruciate ligament (CCLR) is one of the most common causes of hind limb lameness in medium-sized and large dogs (1). It leads to stifle instability, osteoarthritis, and, in 30–80% of cases, to injuries of the medial meniscus (2–4). Various methods have been developed for treatment, including intra- and extracapsular techniques such as tibial plateau leveling osteotomy (TPLO) or tibial tuberosity advancement (TTA) (5). While the cranial cruciate ligament is reconstructed in several intra-articular procedures using various materials, repositioning osteotomies such as TPLO aim to neutralize the cranial tibial thrust caused by CCLR (6–9). In direct comparison with methods such as TTA or extra-articular techniques, TPLO has been found to be the superior method in various studies due to its good results and high owner satisfaction (10–12). However, various studies show that even TPLO cannot guarantee complete stifle stability. Possible postoperative complications with TPLO include femorotibial subluxation and instability (13) as well as secondary meniscus damage (14).

Intra-articular stabilization in dogs with CCLR can be achieved using biological material (over-the-top techniques) or a synthetic ligament replacement (5). Biological reconstruction methods use endogenous tissue to stabilize the stifle joint by replacing the function of the cranial cruciate ligament with parts of the patellar tendon or adjacent structures such as the fascia lata (5). In synthetic ligament replacement methods an artificial ligament is used to replace the cranial cruciate ligament and imitate the mechanical functions of the cranial cruciate ligament (5).

Although anatomical reconstruction is considered to be the gold standard in human medicine, especially for athletes (15, 16), it has not yet become established in veterinary medicine for dogs. This is mainly due to the inability to restore tibial cranial instability and internal rotation (17) as well as an inferior long term outcome when compared to other stabilization techniques (18).

The synthetic ligament marketed by Eickemeyer® as Zlig is made of ultra-high molecular weight polyethylene (UHMWPE) and was already investigated in a similar form in a study by Pagès (19). In this study, 12 of the 15 dogs (80%) showed excellent functional results, and 3 of the 15 dogs (20%) showed good functional results without major complications or implant failure (19). Pagès particularly emphasized the rapid and good weight-bearing capacity (19). In a study by Le Doze et al. (20) involving 101 dogs that underwent surgery using a synthetic ligament replacement, all dogs weighing over 20 kg were able to bear weight on the operated hind leg on the first day after surgery.

Comparative studies between the already established TPLO and the new Zlig procedure are not yet available in the accessible literature. The aim of this study was therefore to investigate whether the intra-articular synthetic ligament replacement technique using a Zlig implant can achieve similar clinical and gait analysis results in cases of CCLR compared to TPLO. Our hypothesis was that dogs whose CCLR is treated with Zlig would achieve comparable or even better results in terms of lameness, limb weight-bearing and pain response than dogs treated with TPLO.

## 2 Materials and methods

This is a prospective, controlled study conducted at the Clinic for Small Animal Surgery and Reproduction at LMU Munich between May 2022 and July 2023. Dogs with a body weight of 20–40 kg and CCLR were included in the study. Exclusion criteria were other orthopedic diseases and previous performed surgeries on the stifle joint less than 4 weeks prior to the study. In dogs that suffered a bilateral cruciate ligament rupture during the course of the study, only the data from the second hind limb was included in the study. The dog owners were given detailed information about both surgical methods and gave their written consent to participate in the study. The choice of which surgical method was used in the patient was then made by the owners themselves.

The Ethics Committee of the Centre for Clinical Veterinary Medicine of Ludwig-Maximilian-University in Munich approved the study protocol (File number 314–23-06-2022).

### 2.1 Preoperative planning and anesthesia

A standard anesthetic protocol was adapted to each patient according to the American Society of Anesthesiologists classification. The standard protocol for premedication consisted of 0.2 mg/kg diazepam (Solupam®, Dechra Veterinary Products Deutschland GmbH, Aulendorf, Germany), 1 mg/kg ketamine (Anesketin, Dechra Veterinary Products Deutschland GmbH, Aulendorf, Germany) followed by propofol according to effect (Narcofol®, CP-Pharma Handelsgesellschaft GmbH, Burgdorf, Germany) were administered for anesthesia induction. This was followed by endotracheal intubation and maintenance of anesthesia with isoflurane (Isofluran CP®, CP-Pharma Handelsgesellschaft GmbH, Burgdorf, Germany). Intraoperatively, the dogs received 10 mL/kg/h of an electrolyte-containing infusion solution (Elektrosel®).

Radiographic examination (X-ray machine Axiom Luminos dRF, Siemens Healthcare GmbH, Erlangen, Germany) of the affected hind limbs was performed in both groups using two orthogonal projections of the stifle joint and a mediolateral projection of the tibia for measuring the tibial plateau angle (TPA) for the TPLO group (8). Dogs in the TPLO group underwent magnetic resonance imaging (MAGNETOM Symphony 1.5 Tesla, Siemens Healthcare GmbH, Erlangen, Germany) to rule out meniscus injury. No MRI examination was performed as standard in dogs in the Zlig group, as the surgical approach for this technique requires an arthrotomy.

### 2.2 Surgical methods

All operations were performed by two experienced orthopedic surgeons (MK and AML). For both surgical methods, the affected stifle was prepared aseptically for the procedure from the hip joint to the tarsal joint.

The TPLO was performed according to the method described by Slocum and Slocum (8), but without the use of a jig. If the clinical investigation or the MRI scan revealed a meniscus injury, a partial meniscectomy of the caudal meniscal horn was performed using a mini-arthrotomy caudal to the medial collateral ligament (21). If there was no evidence of a meniscal lesion, the joint was not opened in dogs in the TPLO group. After placement of the implants, the fit of the plate and the length of the screws were checked using a mobile C-arm (Cios Spin, Siemens Healthineers AG, Forchheim, Germany). The length of the screws was then adjusted as needed. The wound was closed in layers. After cleaning the wound site, a wound dressing was applied to protect the incision. No bandage was applied postoperatively.

Intra-articular ligament replacement using Zlig was performed according to the surgical description on the official Eickemeyer® website (22). First a lateral parapatellar approach to the stifle joint was made followed by an arthrotomy of the stifle joint. If a meniscus injury was detected, a partial meniscectomy of the affected area was performed under visual control. For the ligament replacement, two diagonal bone tunnels were drilled intra-articularly through the femur and tibia along the course of the cranial cruciate ligament. Meanwhile the stifle joint was flexed to the maximum extent to allow isometric placement of the ligament. Two transverse bone tunnels were drilled through the femur and tibia 1–1.5 cm above (femur) or below (tibia) the first tunnel exit. In all cases, a Kirschner drill wire was set first and, once satisfactory placement was achieved, an oversized cannulated drill was used. The bone tunnels were drilled without mechanical targeting aids. In the next step, the Zlig implant was first guided through the two bone tunnels near the joint. After tightening the ligament replacement, it was fixed from proximal to distal using two cannulated interference screws according to the measured length and size. The stability of the stifle joint was then verified by testing for drawer instability. If the result was positive, one screw was loosened and then refixed after retightening the ligament replacement. The ligament was then pulled through the two transverse bone tunnels and secured using two cannulated titanium interference screws, each placed through both cortices. The selection of ligament thickness and screw size was patient-specific, based on a size chart from the official Eickemeyer® website (23). The wound was then closed layer by layer, and a wound dressing was applied. The surgeon rechecked the stability of the stifle joint after wound closure. No bandage was applied.

**Figure 1:**
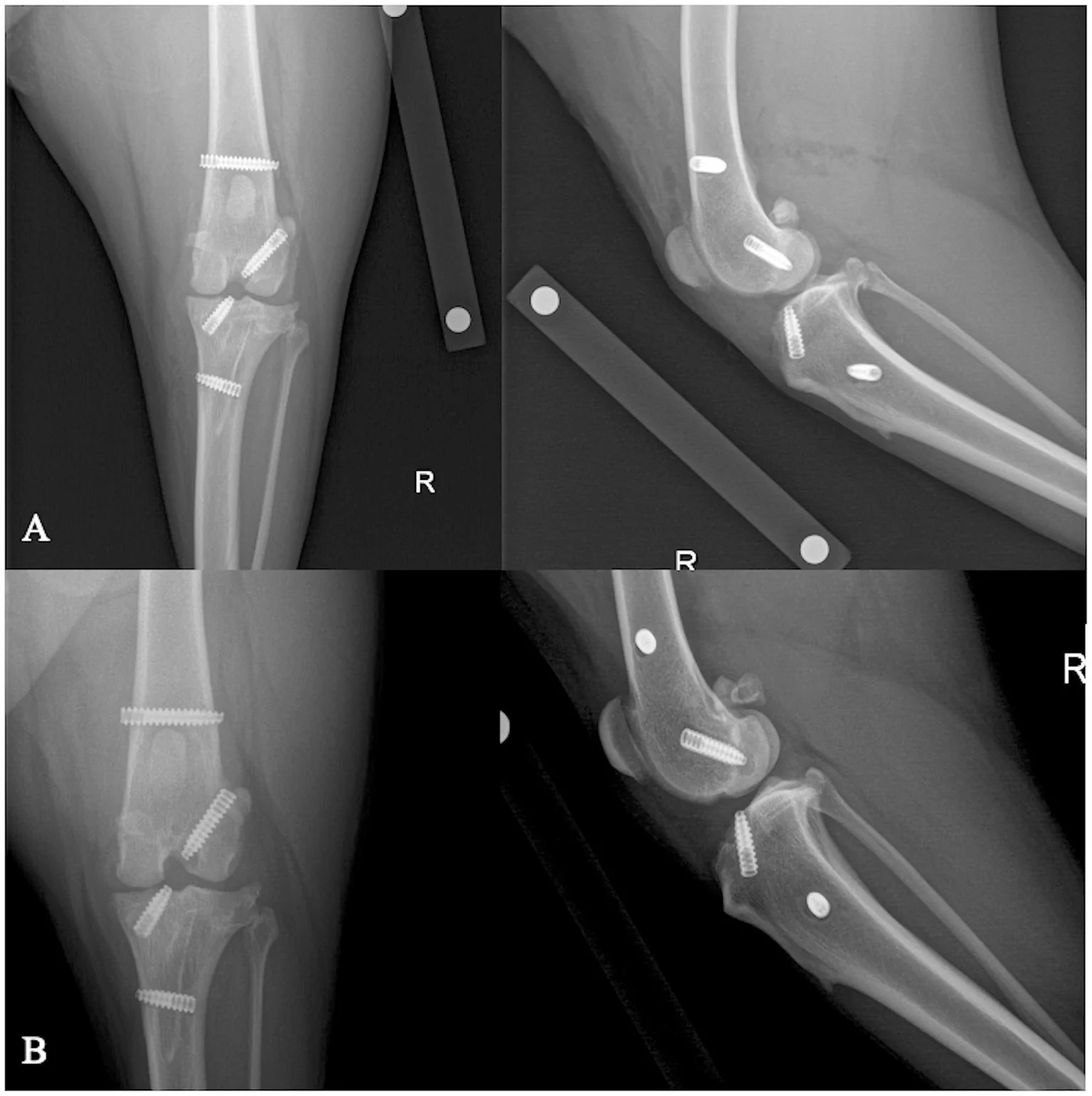
Postoperative mediolateral (right) and caudocranial (left) radiographs of the Zlig procedure. 3-year-old mixed breed (Case 18, first CCLR side, not included in the study). Immediate postoperatively **(A)**, 18 weeks postoperatively **(B)**.

**Figure 2:**
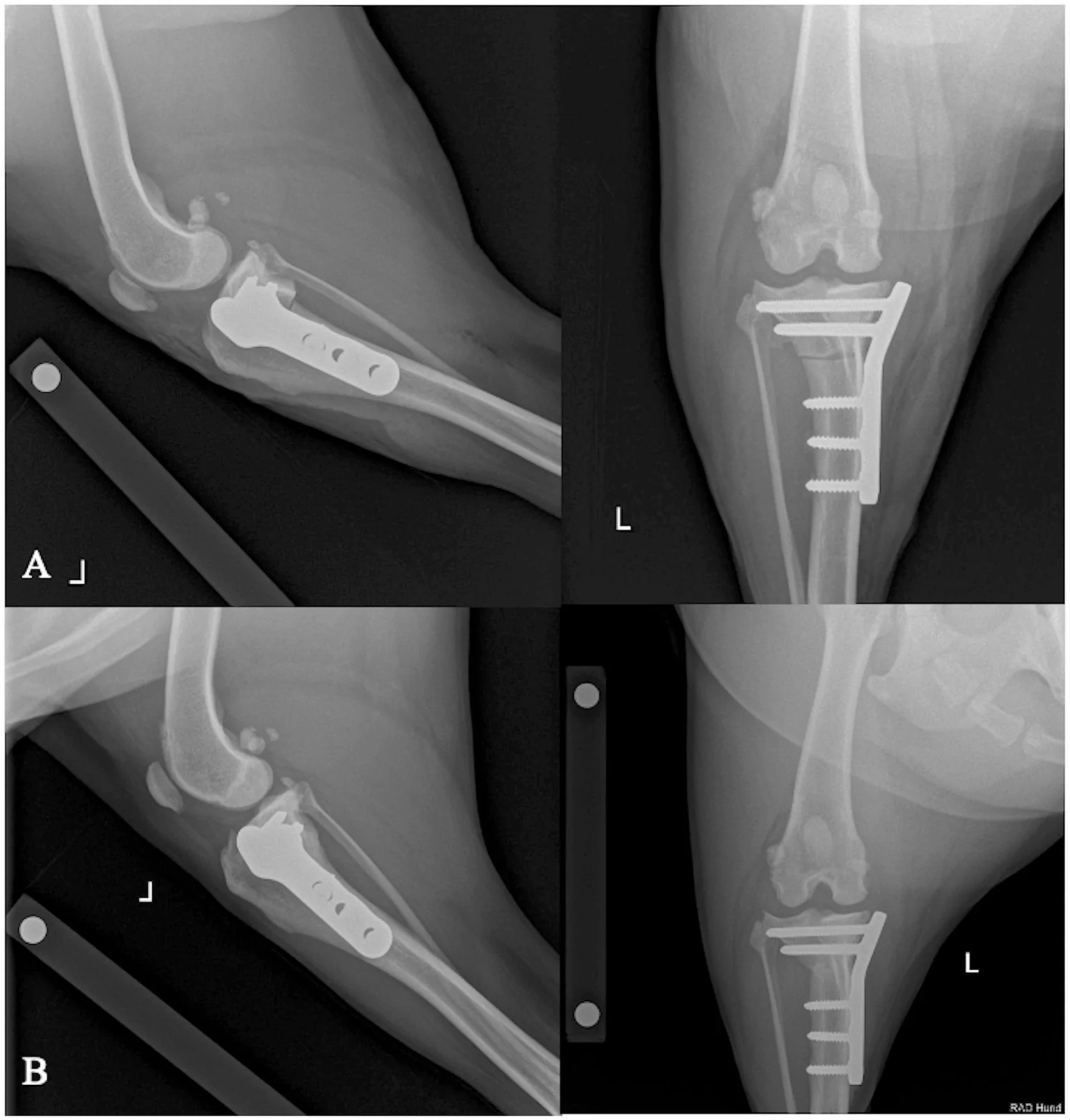
Postoperative mediolateral (left) and caudocranial (right) radiographs of the TPLO procedure. 9-year-old Beagle (Case 2). **(A)** immediate postoperatively, **(B)** 18 weeks postoperatively.

In both methods, postoperative radiographic examination of the stifles in two orthogonal projections was performed (Figures 1, 2). In the TPLO group a mediolateral projection of the tibia was performed for measuring the TPA after surgery.

### 2.3 Intra- and postoperative management

Intraoperatively, all dogs received 0.2 mg/kg methadone (Comfortan®, Dechra Veterinary Products Deutschland GmbH, Aulendorf, Germany). Postoperatively, 0.02 mg/kg buprenorphine (Buprenovet® sine, Bayer Vital GmbH, Leverkusen, Germany) was administered intravenously every 8 h, as well as 4 mg/kg carprofen (Rimadyl®, Zoetis Deutschland GmbH, Berlin, Germany) once daily orally over 7 days. For antibiotic treatment, 20 mg/kg cefazolin (Cephazolin Fresenius 2 g, CP-Pharma Handelsgesellschaft GmbH, Burgdorf, Germany) was administered 30–60 min preoperatively and intraoperatively every 90 min. This was followed by postoperative oral administration of 20 mg/kg cefazolin (Cefatab®, CP-Pharma Handelsgesellschaft GmbH, Burgdorf, Germany) twice daily for further 5 days. The dogs were discharged on average 24 h after surgery. Strict rest was prescribed at home for a period of 8 weeks, with only short walks on a leash permitted.

### 2.4 Follow-up examinations

The two groups were compared based on the results of clinical orthopedic examinations and gait analysis using a treadmill. Radiographs (as previously described) of the affected stifle joints were performed at every follow-up appointment. Data were collected in both groups preoperatively and 6, 12, and 18 weeks postoperatively.

The clinical orthopedic examination assessed subjective lameness (Score 0: no lameness, 1: mild intermittent lameness, 2: mild obvious lameness, 3: moderate lameness, 4: severe lameness), pain during the examination via modified Glasgow Composite Measure Pain Scale (24) (Score 0: no expression, 1: smacking and/or licking, 2: body flinching and/or turning away, 3: growling and/or baring teeth, 4: snapping and/or crying), examination of joint effusion in the stifle joint by palpation (Score 0: no effusion, patellar tendon palpable, 1: mild effusion, slight well-defined palpable bulging of the joint capsule, 2: moderate effusion, palpable non-defined bulging of the joint capsule, 3: severe effusion, tense swelling of the whole stifle joint) as well as the subjective results of the tibial compression test and the drawer test, without measuring the mm (Only positive or negative). The clinical examinations were performed by the same examiner at all times.

The gait analysis was performed at walk on a treadmill with four Kistler force plates (Special elements, German Sport University Cologne, Cologne) and an optical system (Vicon Nexus, Vicon Motion Systems Ltd., Oxford, United Kingdom). The analysis was performed using Quadruped Locomotion Software, which has already been used in other studies (25, 26). The treadmill speed was adjusted individually for each dog in increments of 0.02 m/s and was constant over all measurement times. The peak vertical force (PVF) and vertical impulse (VI) were evaluated, each as a percentage of the body weight. The symmetry indices of the hind limbs for PVF (SIPVF) and VI (SIVI) were calculated according to the method described by Voss et al. (27). A threshold value of SIPVF < 9% at the last check-up 18 weeks postoperatively was considered objectively free of lameness (27). Postoperative minor and major complications were recorded and categorized based on the classification by Cook et al. (28).

### 2.5 Statistical analysis

Due to the presence of repeated measures, the primary outcome variables (PVF, VI, SIPVF, and SIVI) and the secondary variables (degree of lameness and joint effusion) were analyzed using mixed-effects models (MEMs), with individual animals included as a random effect and the interaction between group and time included as fixed effects. The following model assumptions were assessed in all cases: (1) normality of residuals using the Shapiro–Wilk test, (2) homogeneity of variances between groups using Bartlett’s test, and (3) homoscedasticity (constant error variance) using the Breusch–Pagan test. When assumptions were satisfied, linear mixed-effects models were used (R package: lmer). When assumptions were violated, robust linear mixed-effects models were applied (R package: robustlmm). In addition, linear and robust linear models were compared using the following performance indicators: conditional coefficient of determination (*R*^2^), marginal coefficient of determination (*R*^2^), intraclass correlation coefficient (ICC), and root mean square error (RMSE). The model showing the best overall fit was selected.

The variables pain during manipulation, cranial drawer test, and tibial compression test were initially intended to be evaluated using mixed-effects logistic regression. However, due to the small number of positive events, these models did not converge. Therefore, a Bayesian logistic regression model without a random effect was used instead. Age was compared between groups using Student’s *t*-test because the data were normally distributed and showed homogeneous variance. Body condition score (BCS) was compared between groups using the nonparametric Mann–Whitney *U* test.

All contrasts (differences) between groups were assessed after model fitting using estimated marginal means (R package: emmeans), with Tukey-adjusted *p*-values for multiple comparisons. Results with a *p* value <0.05 were considered statistically significant. Data analysis was performed using R 4.5.0 (2025-04-11).

## 3 Results

A total of 30 dogs (TPLO group *n* = 16); Zlig group *n* = 14) were included in the study (Tables 1, 2). The mean age was 6.3 years in the TPLO group and 5.8 years in the Zlig group. The mean age did not differ significantly between the groups (*p* = 0.0994). The mean weight was 31.3 kg in the TPLO group and 25.4 kg in the Zlig group. The BCS averaged 5 in both groups. Dogs in the TPLO group had a higher mean weight than dogs in the Zlig group (*p* = 0.0019). Most dogs in the TPLO group were mixed breeds (*n* = 5), followed by Labrador Retrievers (*n* = 5). Other breeds were represented only once (Table 1). In the Zlig group, mixed breeds were also the most common (*n* = 6), followed by two Beagles and two Boxers. Other breeds were represented only once (Table 2).

**Table 1:** Patient information and results after 18 weeks in the TPLO group (m, male; mn, male neutered; f, female; fn, female neutered, y, years).

Nr	Surgery	Breed	Sex	Age	Weight in kg	BCS	CCLR side (included in the study)	Meniscal injury	Complications	Lameness degree at 18 weeks
1	TPLO	German shorthaired Pointer	m	10y	35.2	5/9	right	yes	—	0
2	TPLO	Beagle	m	9.1y	21.3	8/9	left^a^	yes	—	0
3	TPLO	Mixed breed	fn	3.3y	27.2	5/9	left^a^	no	—	0
4	TPLO	Labrador Retriever	m	5.1y	35.2	8/9	left	yes	—	0
5	TPLO	Mixed breed	mn	4.9y	29.7	5/9	right	no	—	0
6	TPLO	Mixed breed	mn	3.4y	32.5	5/9	right	no	Superficial wound healing disorder	1
7	TPLO	Rottweiler	mn	7.7y	39.6	7/9	left^a^	no	—	0
8	TPLO	Labrador Retriever	m	8.4y	32	5/9	left^a^	no		0
9	TPLO	Border Collie	m	10.6y	20	4/9	right	yes	—	3
10	TPLO	Continental Bulldog	fn	6.2y	30.2	8/9	left	no	—	0
11	TPLO	Mixed breed	mn	6.9y	31.6	6/9	right^a^	yes	—	0
12	TPLO	Mixed breed	fn	8.3y	25.6	5/9	right^a^	yes	—	0
13	TPLO	Dobermann	f	1.8y	30	4/9	both sides (right)	yes	Superficial wound healing disorder	0
14	TPLO	Golden Retriever	f	1.3y	25.3	5/9	both sides (right)	no	Superficial wound healing disorder	0
15	TPLO	Beauceron	f	7.9y	35.4	5/9	left	no	—	0
16	TPLO	Labrador Retriever	mn	12.9y	40	8/9	right^a^	yes	—	1

^a^Dogs with TPLO in the stifle joint of the contralateral limb more than 6 months ago.

**Table 2:** Patient information and results after 18 weeks in the Zlig group (m, male; mn, male neutered; f, female; fn, female neutered, y, years).

Nr	Surgery	Breed	Sex	Age	Weight in kg	BCS	CCLR side (included in the study)	Meniscal injury	Implant size	Complications	Lameness degree at 18 weeks
17	Zlig	Beagle	m	6.4y	23	7/9	left	yes	32/17, 4.0 mm, 4.5 mm	—	0
18	Zlig	Mixed breed	fn	3.3y	25	4/9	both sides (left)	no	48/19, 4.5 mm	Late meniscal injury with wound healing disorder after second surgery^a^	0
19	Zlig	American Bulldog	fn	6.7y	38	7/9	right	yes	48/22, 4.5 mm	Superficial wound healing disorder	1
20	Zlig	Mixed breed	f	9.4y	24.4	6/9	left	yes	48/19, 4.0 mm	—	0
21	Zlig	Mixed breed	fn	3.1y	24.4	4/9	both sides (right)	yes	32/17, 4.0 mm	—	0
22	Zlig	Malinois	mn	10.9y	24	4/9	right	yes	32/17, 4.0 mm	—	1
23	Zlig	Mixed breed	fn	10y	21.3	5/9	left	no	32/17, 3.5 mm	—	3
24	Zlig	Old English Bulldog	f	1.1y	26	6/9	right	no	32/17, 3.5 mm, 4.0 mm	—	1
25	Zlig	Boxer	f	0.8y	24	4/9	left	yes	initial surgery: 32/17, 4.0 mm; revision: 48/19, 4.0 mm, 4.5 mm	Implant failure (complete rupture of the ligament)	1
26	Zlig	Mixed breed	fn	2.7y	30.5	5/9	both sides (left)	yes	32/17, 4 mm	—	0
27	Zlig	Border Collie	f	8.2y	20	4/9	left	yes	initial and revision surgery: 32/17, 3.5 mm, 4.0 mm	Implant loosening	4
28	Zlig	Boxer	f	6.9y	25.5	5/9	left	no	32/17, 4.0 mm	At week 6: lameness and pain during manipulation	1
29	Zlig	Mixed breed	m	9.4y	21	6/9	left	yes	32/17, 4.0 mm	—	0
30	Zlig	Beagle	m	7.2y	23	7/9	right	no	32/17, 4.0 mm	—	1

^a^Dog with intraoperative femoral fissure.

In the TPLO group, 7/16 dogs already had a TPLO-treated CCLR on the contralateral limb at the time of presentation, which had occurred at least 6 months previously. One dog in the TPLO group developed a bilateral CCLR during the course of the study. In this dog both stifle joints were treated with TPLO within 4 months and only the stifle with second surgery was included in the study (Table 1).

In the Zlig group, none of the dogs had previously undergone knee surgery. However, three of the 14 dogs developed bilateral cruciate ligament rupture during the course of the study. In these cases, both limbs were surgically treated with Zlig within 2–4 months and only the data from the second surgery was included in the study (Table 2).

### 3.1 Surgical procedure

In all dogs in the TPLO group, the surgery proceeded without any further intraoperative complications. Overall, the meniscus was injured in 8/16 stifle joints, and the caudal horn of the medial meniscus was removed (Table 1) via mini-arthrotomy (21). In one patient, after checking the implants using a C-arm, one screw was subsequently replaced with a shorter one. On the postoperative X-rays, a tibial plateau angle (TPA) of 2–6° was achieved in all stifle joints. In all joints, the postoperative tibial compression test was negative.

**Figure 3:**
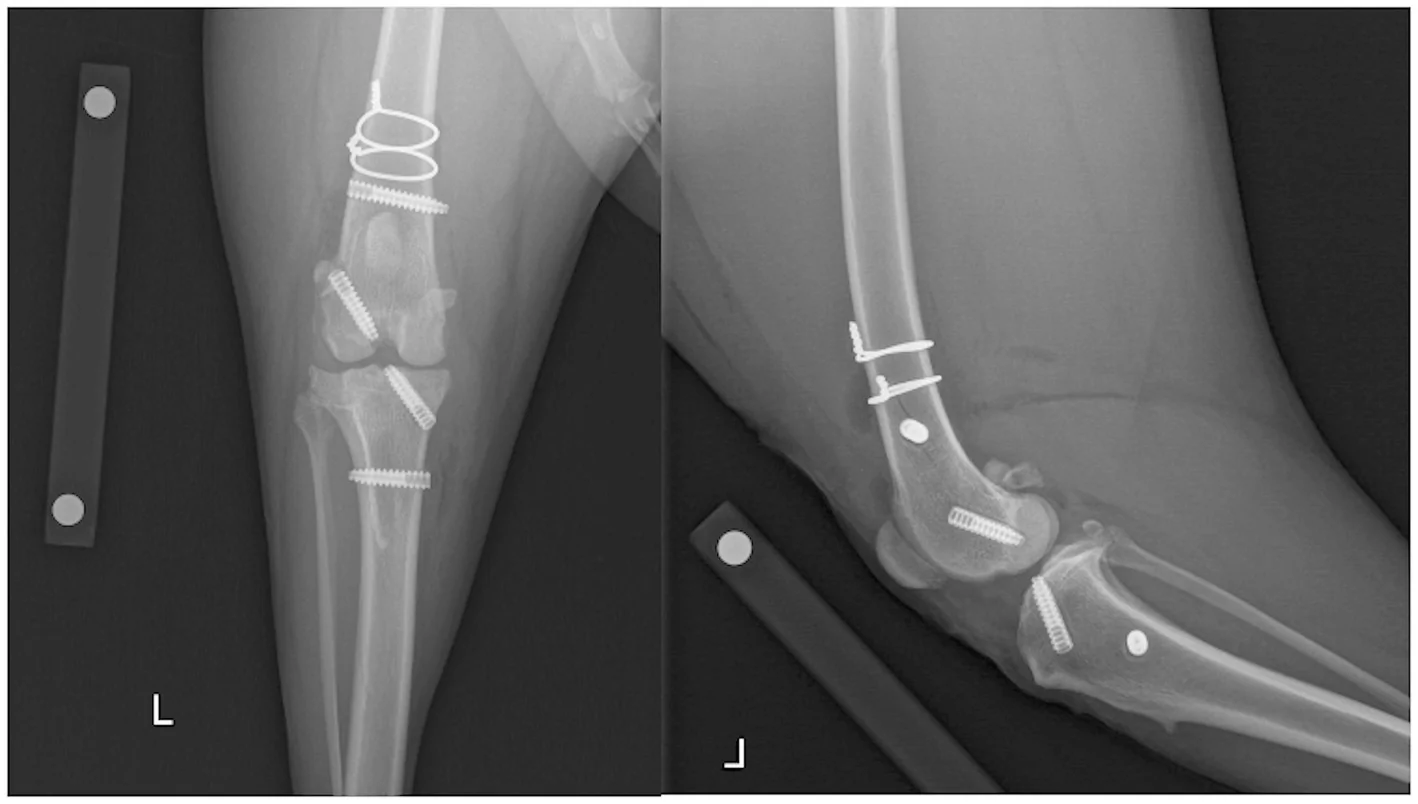
Postoperative mediolateral (right) and caudocranial (left) radiograph of a 3 year old mixed breed dog (Case 18, second CCLR side, included in the study) with an intraoperative femoral fissure treated with two cerclage wires.

In the dogs of the Zlig group, the caudalhorn of the medial meniscus was injured in 7/14 dogs (Table 2) and was removed during surgery. In one dog (Case 18), a transcortical femoral fissure occurred during placement of the proximal screw. The fissure was confirmed using a mobile C-arm (Cios Spin, Siemens Healthineers AG, Forchheim, Germany) and did not extend into the joint. It was fixated using two wire cerclages (Figure 3). The drawer test was negative in all cases immediate postoperatively.

### 3.2 Postoperative complications

No major complications occurred in the TPLO group.

In the Zlig group, three major complications were observed in a total of 3 of the 14 dogs (21.4%). One case (Case 18) of postoperative meniscus injury that was diagnosed after 16 weeks postoperatively (Table 2). The caudal part of the medial meniscus was removed via a medial mini-arthrotomy and this dog developed wound dehiscence with suspected ligament infection that required surgical revision with partial implant removal. In this joint, the proximal tibial screw was replaced with a larger screw, and the distal tibial screw, along with the distal ligament, was removed. No further complication occurred in this dog. A second dog (Case 25) was presented with severe lameness 9 weeks postoperatively due to complete ligament rupture (Table 2). A larger ligament was inserted in a subsequent operation. In the third dog (Case 27), ligament loosening was detected 12 weeks postoperatively (Table 2) and the ligament was tightened more securely and fixed again in a second operation with new screws of the same size as before. Both dogs subsequently showed continuous improvement in weight-bearing capacity up to the final follow-up visit (Table 2).

Three minor postoperative complications were observed in the TPLO group (Case 6, 13, 14). In all cases, these were partial wound dehiscences/superficial wound infection that healed completely under oral antibiotic therapy with 20 mg/kg cefazolin (Cefatab®, CP-Pharma Handelsgesellschaft GmbH, Burgdorf, Germany) every 12 h for 5 days. The overall rate of minor postoperative complications was 18.8%.

Two minor postoperative complications were observed in the Zlig group. One dog (Case 19) developed a partial wound dehiscences/superficial wound infection, which was treated with local wound care and oral administration of 12.5 mg/kg amoxicillin-clavulanic acid (Amoxiclav®, CP-Pharma Handelsgesellschaft GmbH, Burgdorf, Germany) every 12 h for 7 days. One other dog (Case 28) was presented 6 weeks postoperatively with persistent lameness and pain in the operated hind limb of unknown origin, which improved with oral administration of 1 mg/kg robenacoxib (Onsior®, Elanco Deutschland GmbH, Monheim, Germany) every 24 h and resting for 7 days. After about 2 weeks, the lameness had almost completely disappeared without the need for medication.

An overview of the documented minor and major complications in both groups is shown in Table 3. The overall complication rate was 18.8% (3/16) in the TPLO group and 35.7% (5/14) in the Zlig group.

**Table 3:** Overview of minor and major postoperative complications for the TPLO and the Zlig group.

Complication	Minor complications		Major complications	
Surgery	TPLO	Zlig	TPLO	Zlig
Implant loosening	—	—	—	1
Implant failure (complete rupture of the ligament)	—	—	—	1
Late meniscal injury with secondary wound healing disorder	—	—	—	1
Superficial wound healing disorder	3	1	—	—
Persistent lameness and pain during manipulation	—	1	—	—

### 3.3 Clinical examination

All dogs in both groups showed improvement in lameness, joint effusion, and pain during manipulation over the study period of 18 weeks.

The degree of lameness decreased significantly in both groups from preoperatively to 18 weeks postoperatively [TPLO group: Estimated change 2.06 points (95% CI: 1.39 to 2.73, *p* < 0.0001); Zlig group: estimated change 1.87 points (95% CI: 1.15 to 2.60, *p* < 0.0001)]. A significant difference between the groups was observed in week 12 in favor of the TPLO group (Δ = −0.96 points, 95% CI: −1.66 to −0.26; *p* = 0.0073). At the all other time points, there were no significant differences between the TPLO group and the Zlig group. At the final follow-up, in the TPLO group 2/16 dogs showed grade 1 lameness and 1/16 dogs showed grade 3 lameness (Table 1). 13/16 (81.3%) dogs were considered subjectively free of lameness. In the Zlig group 6/14 dogs showed grade 1 lameness, 1/14 dogs showed grade 3 lameness and 1/14 dogs (case 11; dog with a loosened ligament after 12 weeks) showed grade 4 lameness (Table 2). 6/14 (42.9%) dogs were considered subjectively free of lameness 18 weeks postoperatively.

Both groups showed a significant reduction in joint effusion in the clinical examination during the study [TPLO group: Estimated change 1.25 (95% CI: 0.83 to 1.66, *p* < 0.0001); Zlig group: estimated change 0.86 (95% CI: 0.42 to 1.3, *p* < 0.0001)]. A significant group difference was observed in week 12 (Δ = −0.55, SE = 0.19; *p* = 0.0050) and week 18 (Δ = −0.52, SE = 0.19; *p* = 0.0075), in each case in favor of the TPLO group. There were no significant differences at the other time points.

The pain response to manipulation of the stifle joint decreased significantly in both groups by week 18 postoperatively. In the TPLO group, the proportion of dogs with pain decreased from preoperatively (mean: 0.936; 95% CI 0.741 to 1.129) to week 18 (mean: 0.250; 95% CI: 0.051 to 0.441; *p* < 0.001). In the Zlig group, there was also a significant decrease between preoperative (mean: 1.002; 95% CI: 0.788 to 1.206) and week 18 (mean: 0.501; 95% CI: 0.292 to 0.711; *p* < 0.001). In the group comparison, there were no significant differences in pain response at any of the time points examined.

Because TPLO does not aim to eliminate positive drawer test, the postoperative findings of this test were not analyzed. In the Zlig group, there was a decrease from 100% (14/14) preoperatively (mean: 0.999; 95% CI: 0.761 to 1.225) to 78.6% (11/14) in week 18 (mean: 0.786; 95% CI: 0.552 to 1.019), which was not significant (*p* = 0.129).

The number of dogs with a positive tibial compression test decreased in both groups up to week 18 postoperatively. In the TPLO group, the number of dogs tested positive decreased from 81.3% (13/16) preoperatively (mean: 0.814; 95% CI: 0.659 to 0.961) to 0% (0/16) 18 weeks postoperatively (mean: 0.001; 95% CI: −0.155 to 0.153; *p* < 0.001). In the Zlig group, there was a decrease from preoperatively (100%; 14/14; mean: 1.000; 95% CI: 0.834 to 1.158) to week 18 (78.6%; 11/14; mean: 0.786; 95% CI: 0.628 to 0.949), which was not significant (*p* = 0.069). Preoperatively, there was no significant difference between TPLO and Zlig. There were significant differences between the groups at all postoperative time points in favor of the TPLO group (week 6: *p* < 0.001; week 12: *p* < 0.001; week 18: *p* < 0.001).

**Figure 4:**
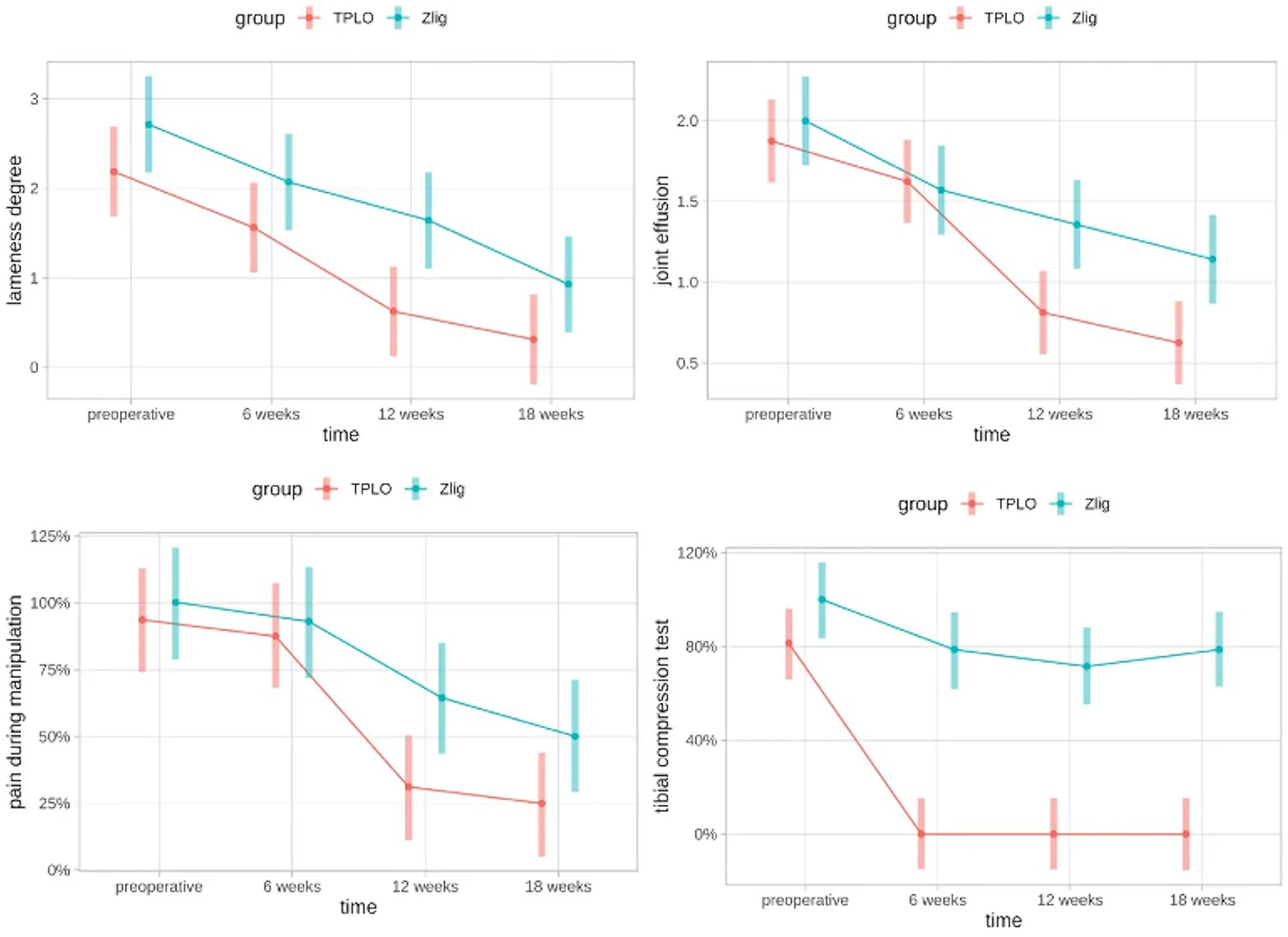
Results of the linear mixed effect models for clinical examination outcome (lameness degree, joint effusion, pain during manipulation, Tibial Compression Test) for both groups from preoperative to 18 weeks postoperatively. The results for the TPLO group are shown in red, and those for the Zlig group in turquoise.

Figure 4 shows the results of these models.

### 3.4 Gait analysis

The PVF improved significantly in both groups from preoperatively to 18 weeks postoperatively [TPLO group: estimated change 4.34 (95% CI: −8.43 to −0.24, *p* = 0.0332); Zlig group: estimated change 5.42 (95% CI: −9.80 to −1.04, *p* = 0.0080)]. There was a significant difference between the groups at week 6 postoperatively in favor of the TPLO group (estimated difference: 4.96; 95% CI: 0.86 to 9.07; *p* = 0.0177). There were no significant differences between the groups at the other time points.

The vertical impulse (VI) increased in both groups from preoperatively to 18 weeks postoperatively [TPLO group: estimated change 0.13 (95% CI: −1.81 to 1.54; *p* = 0.9970); Zlig group: estimated change 1.40 (95% CI: −3.19 to 0.39; *p* = 0.1844)]. There were no significant differences between the groups at any time point.

The symmetry index of peak vertical force (SIPVF) improved significantly in both groups from preoperatively to 18 weeks postoperatively [TPLO group: estimated change 25.33 (95% CI: 8.08 to 42.6; *p* = 0.0013); Zlig group: estimated change 20.16 (95% CI: 1.73 to 38.6; *p* = 0.0264)]. There were no significant group differences at the time points examined. At the last check-up 18 weeks postoperatively, 6/16 of the TPLO dogs and 4/14 of the Zlig dogs were considered objectively free of lameness (SIPVF <9%).

The symmetry index of the vertical impulse (SIVI) improved significantly in both groups from preoperatively to 18 weeks postoperatively [TPLO group: estimated change 24.84 (95% CI: 6.44 to 43.2; *p* = 0.0029); Zlig group: estimated change 24.34 (95% CI: 4.68 to 44.0; *p* = 0.0080)]. There were no significant group differences at the time points examined.

**Figure 5:**
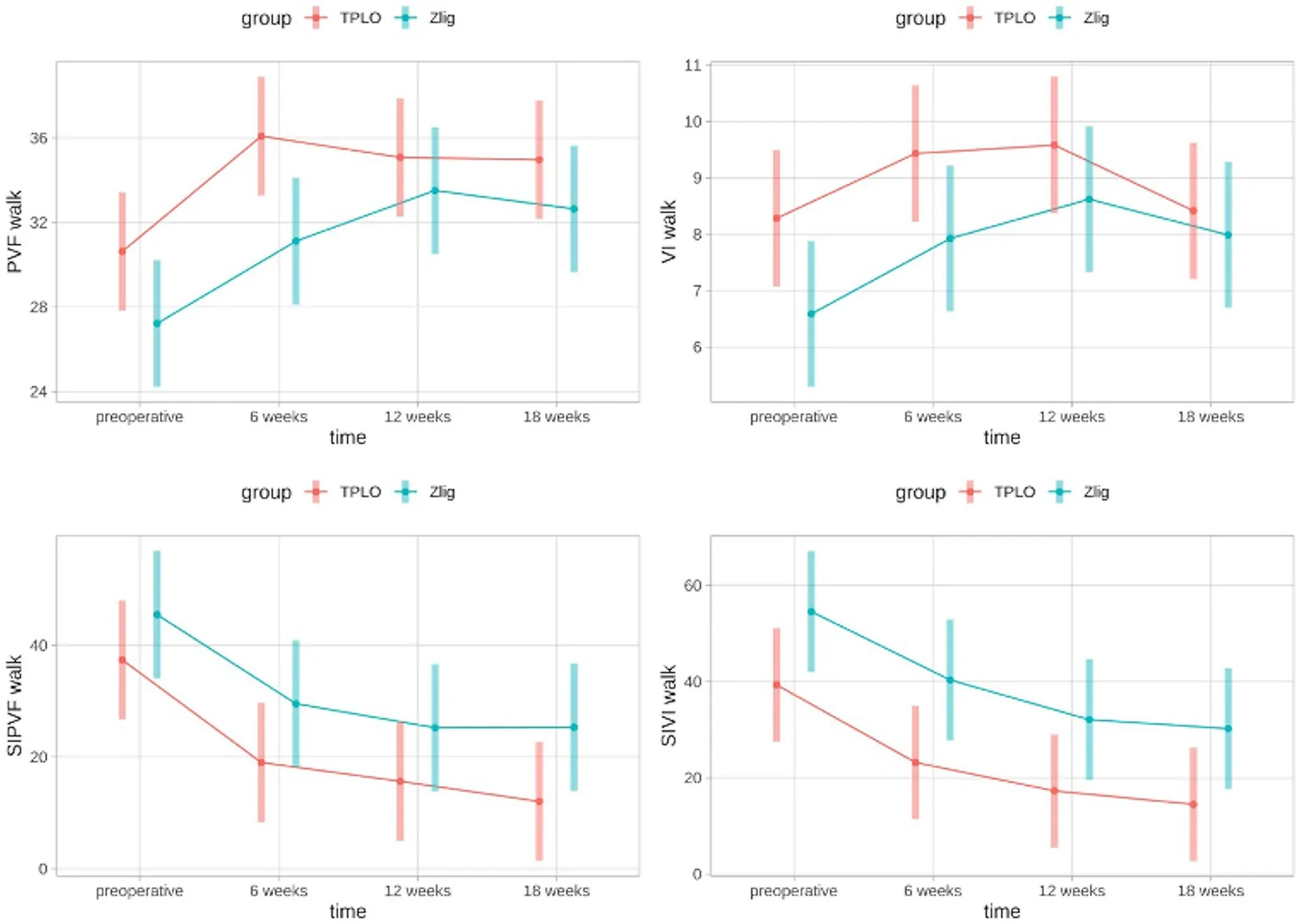
Results of the linear mixed effect models for ground reaction forces (PVF, VI, SIPVF, SIVI) for both groups from preoperative to 18 weeks postoperatively. The results for the TPLO group are shown in red, and those for the Zlig group in turquoise.

Figure 5 shows the results of these models.

## 4 Discussion

The aim of this prospective study was to conduct an objective, clinical-functional comparison of TPLO with the intra-articular synthetic ligament replacement Zlig in dogs with CCLR. The hypothesis that Zlig would result in comparable or better outcome regarding lameness, limb weight-bearing and pain response than dogs treated with TPLO was only partially supported.

During the clinical examination, both groups showed significant improvements in terms of lameness, joint effusion, and pain during manipulation. The degree of lameness decreased significantly in both groups until the last check-up after 18 weeks. Similar results for TPLO were also reported by Volz et al. (25) and Knebel et al. (26) who described a continuous improvement in lameness scores up to 6 months postoperatively. In a study by Krotscheck et al. (29), dogs that had undergone TPLO surgery returned to a normal gait after 150 days. Regarding intra-articular ligament replacement Ödman et al. (30) described a return to normal gait pattern just 1 month postoperatively after using an UHMWPE implant. This observation could not be confirmed in this study. At the 6-week follow-up, 10/14 dogs in the Zlig group still had a lameness score of 2 or higher. However, even in the TPLO group, 9/16 dogs still had grade 2 or higher lameness after 6 weeks. Nevertheless both groups showed a significant improvement in lameness by the final follow-up at 18 weeks (Figure 4).

In the present study, a significant difference in favor of the TPLO group was only found regarding the reduction of joint effusion at the last follow-up after 18 weeks. The other parameters of the clinical examination did not show any notable differences between the groups. Nevertheless, it was noticeable that all three clinical parameters showed a greater improvement in the TPLO group than in the Zlig group at the 12-week check-up. The rapid recovery after intra-articular ligament replacement described by Le Doze et al. (20) compared to TPLO could not be confirmed in the present study.

In the TPLO group, all dogs showed a negative tibial compression test after 18 weeks, indicating successful neutralization of cranial tibial thrust, which is the goal of TPLO (8). In the Zlig group, the test remained positive in 78.6% (11/14) of dogs at the last check-up, despite this test being negative immediate postoperatively. An ex vivo study by Lampart et al. (31) evaluating the accuracy of three manual laxity tests for cranial cruciate ligament rupture, showed that even in intact cranial cruciate ligaments, a slight translation could be induced. These findings, along with stifle instability described in various studies using an UHMWPE implant for intra-articular ligament replacement (30, 32), may potentially explain the high number of stifle joint instability observed in our study. Again, it should be noted that in the present study, the tibial compression test and the drawer test was recorded as either positive or negative, and it cannot be said with certainty how relevant this was for the joint.

While cranial cruciate ligament replacement techniques should provide direct stabilization until the ligament structures are incorporated into the endogenous tissue and periarticular fibrosis takes over the necessary stability (33), this was not seen in the current study. The cranial drawer test remained positive in 78.6% (11/14) of the dogs in the Zlig group after 18 weeks. Ödman et al. (30) described in their case report about the use of an UHMWPE implant as an intra-articular ligament replacement a slight, persistent instability of the stifle after 6 months, which had no clinical impact. This was confirmed in the present study, as all dogs in the Zlig group showed improvement in the subjective lameness examination and in the objective gait analysis over the 18-week study period. However, in a study by Barnhart et al. (32), an increase in dogs with stifle instability was described over the course of the 24-week study, which was also associated with higher degrees of lameness. In Barnhart et al. (32), 16.7% (7/42) of dogs that received synthetic ligament replacement showed a positive cranial drawer test in week 4, compared to 42.9% (18/42) of positive dogs at the 24-week check-up. The authors attribute this high number to factors such as incomplete tissue ingrowth, inadequate strength of the initial fixation, tunnel abrasion or failure of the synthetic ligament (32). In the present study, no second-look arthroscopy was performed to assess the integration of the Zlig ligament replacement. It therefore remains unclear whether implants that were no longer intact were responsible for the persistent instability of the stifle joints and whether the dogs in this study with a positive drawer sign have developed capsular fibrosis.

In the present study, the stability of the intra-articular ligament replacement was tested before the end of the operation. However, tunnel abrasion or ligament failure may still be potential causes of a positive drawer test. Goin et al. (34) showed with a biomechanical cyclic loading test of an UHMWPE implant for the treatment of cranial cruciate ligament rupture in dogs, that two of the seven specimens exhibited a displacement of >3 mm at the end of the dynamic loading and were therefore classified as “defective.” This finding may correspond to the clinically detectable displacement observed during the cranial drawer test in the present study after 18 weeks of gradually increasing stress on the limb. However, this study did not specify by how many millimeters the drawer test could be triggered. In an ex vivo biomechanical study by Sun et al. (17) on combined extra- and intracapsular stabilization in dogs, the internal rotation caused by a cranial cruciate ligament rupture could not be neutralized by an intracapsular reconstruction alone. The authors cite poor graft stiffness and fixation strength as possible causes (17). The present study did not include a specific assessment of internal rotational instability or pivot shift. Therefore, no conclusions can be drawn regarding rotational stability following either of the two surgical procedures which reflects a limitation of the current study. Internal rotational instability is considered a major cause of the pivot-shift phenomenon (35). Persistent instability of the stifle joint can contribute to meniscal injuries, cartilage damage, and the progression of osteoarthritis (35). Future studies evaluating the Zlig implant should therefore include a quantitative assessment of internal rotational instability, as well as an evaluation of meniscal injuries, the progression of osteoarthritis and the integrity of the implant over an extended period of time.

Gait analysis confirmed functional improvement in both groups. Significant group differences were only observed preoperatively and at week 6 in favor of the TPLO group. At the 6-week follow-up, PVF was significantly higher in the TPLO group than in the Zlig group, indicating greater load on the operated limb during the initial recovery phase. This might indicate rapid functional recovery of the operated limb in the TPLO group at 6 weeks postoperatively.

Subjective visual lameness assessment and force plate analysis are used to evaluate various aspects of limb function. Subjective lameness assessment is used to identify clinically detectable gait abnormalities, while objective gait analysis quantifies irregularities in the PVF on the limbs (36). Low agreement between subjective and objective gait analysis has already been described in the literature (36, 37). In a study by Waxman et al. (37), the results of subjective gait assessments by experienced and inexperienced observers were compared with the objective results of gait analysis. The subjective assessment of gait varied among the evaluators and correlated only weakly with objective measures. This concurs with our finding that there was a discrepancy between subjective lameness scoring and objective gait analysis at the 18-week follow-up. After 18 weeks, 62.5% (10/16) of the TPLO dogs and 71.4% (10/14) of the Zlig dogs were considered objectively lame according to the classification by Voss et al. (27). In contrast to the objective gait analysis, the subjective lameness assessment at 18 weeks found that 3/16 (18.7%) dogs in the TPLO group and 8/14 (57.1%) dogs in the Zlig group were considered subjectively lame. The results of the study suggest that the observed clinical improvement in lameness in both groups cannot bei interpreted as a complete recovery of limb function. In the study by Volz et al. (25), 24.4% (10/41) of the TPLO dogs were considered objectively lame at the 6-month follow-up. The poorer results in the present study could possibly be explained by the shorter observation period of only 18 weeks. Since no significant differences were observed between the two groups at the 18-week follow-up, neither surgical technique can be considered superior to the other.

The overall postoperative complication rate in this study of 18.8% (3/16) in the TPLO group is consistent with other findings in the literature. For example, Volz et al. (25) reported an overall complication rate of 17.1%, and Schuenemann and Kaczmarek (38) reported a rate of 22% in large dogs. 35.7% (5/14) of the dogs in the own Zlig group experienced a complication, including three dogs with a total of 3 major complications and one complication following the surgery for the first complication. Barnhart et al. (32) reported complication rates of 50% (25/50) in their study about the use of a synthetic intra-articular ligament replacement, including 16 major complications. The most common major complication observed here was a ligament rupture, which occurred either alone or in combination with other complications (medial patellar luxation, medial meniscal injury). The major complications that occurred in the present study were also a ligament rupture, ligament loosening, secondary meniscal injury, and a wound infection with suspected implant infection.

The two dogs in our own study that required revision surgery due to implant rupture (*n* = 1) and implant loosening (*n* = 1) were both large (>20 kg), very active dogs. In the first case (implant rupture), the dog weighed 24 kg and was therefore suitable for both a smaller and a larger implant according to Eickemeyer’s size chart. During surgery, the decision was made to use the thinner Zlig ligament and thinner screw size, which are also listed in the Zlig reference table for this weight. During the revision surgery, the thicker ligament, which was also suitable according to the size chart and larger screws were implanted. In the second case, a suitable implant was also selected according to the Zlig reference table. However, implant loosening occurred and new screws of the same size were used for fixation during the second surgery. Future studies should investigate the extent to which the Zlig implant is suitable for active dogs weighing over 20 kg and the criteria by which implants should be selected.

Intraoperatively, one patient in the Zlig group developed a diaphyseal femoral fissure, which was fixed using two cerclage wires. The fissure originating from the transcortical region occurred during placement of the proximal transverse femoral screw. Possible causes could include the use of non-self-tapping screws, the insertion of the interference screw into the trans-cortical region, and the resulting expansion of the bone. This did not result in any further complications, and the dog showed no further abnormalities during the postoperative healing process. The fissure was still radiographically visible at the final follow-up appointment after 18 weeks, although less clearly than immediately after surgery. In a study by Pfund and Wendelburg (39) on THR in dogs, 9.8% (22/224) of the dogs had intraoperative fissures or fractures (21 fissures and one fracture), which in most cases were associated with broaching. 93% (200/215) of the dogs showed a complete return to normal weight-bearing within 1 year (39). No follow-up data was available for the 9 missing dogs. In the study by Barnhart et al. (32), transcortical tibial fractures were detected in 2/50 patients during postoperative radiographic follow-up, which had completely healed after 24 weeks.

Postoperative meniscal damage was documented in only one patient (1/7) in the Zlig group at 16 weeks and none in the TPLO group. This percentage of 7.1% (1/15) of dogs in the own study with postoperative meniscal damage is consistent with other studies, which describe an incidence of 6.3–13.8% depending on the surgical method (40, 41). Case et al. (42) described in their study of 26 dogs that underwent second-look arthroscopy following initial surgical treatment of their CCLR using various surgical techniques (TPLO, lateral suture fixation, TTA, tensor fascia lata graft) that the majority of such lesions were diagnosed after an average of 5 months. The association between persistent cranial cruciate ligament insufficiency and meniscal injury has also been described by Flo (43). The persistent stifle joint instability of dogs in the Zlig group may represent a possible risk factor for postoperative meniscal injury. Still, a misidentified meniscal lesion during initial surgery cannot be ruled out for the case in this study. Regarding the time frame for the occurrence of postoperative meniscal lesions reported by Case et al. (42), the present study may underestimate the incidence of postoperative meniscal tears. Longer-term studies with a quantitative assessment of stifle instability are required in the future.

The present study has several limitations. The small number of study patients in both groups should be viewed critically when compared to other studies on cranial cruciate ligament ruptures in dogs (25, 26, 29). No sample-size calculation was performed which is increasing the risk of type II error in the study groups.

Dogs with bilateral CCLR were also included in the study (TPLO *n* = 1, Zlig *n* = 3). Although only the most recently operated limb was included in the evaluation, it cannot be ruled out that the gait pattern was influenced by the other hind limb, which was also impaired. The reason for including dogs with bilateral cruciate ligament rupture was to achieve a larger number of study participants. The second reason was to account for the high number of dogs with bilateral cruciate ligament rupture in the overall population (44), as only a few prospective studies on unilateral cruciate ligament rupture have been published at the time of this study (45). It should also be noted that the surgeons in this study had little experience with intra-articular replacement method Zlig, but in contrast had many years of experience with the TPLO surgical procedure.

In this study, the postoperative stability of the stifle joint was only assessed clinically using drawer test and tibial compression test. The drawer test was recorded as either positive or negative and no specific assessment of internal rotational instability or pivot shift was performed. Furthermore, there was no objective postoperative mechanical instability assessment using radiographic measurements of cranial tibial translation. A more detailed objective or subjective clinical and radiographic examination would be particularly useful in terms of assessing the progression of instability and the possible future consequences.

The present study showed that both TPLO and intra-articular Zlig ligament replacement led to a significant subjective and objective improvement in dogs with CCLR within a period of 18 weeks. Dogs that underwent Zlig ligament replacement surgery showed a higher rate of complications and a higher number of postoperative unstable stifle joints in this study compared to dogs that underwent TPLO. The residual instability in dogs treated with Zlig requires further investigation such as biomechanical ex vivo studies to better identify the forces acting on the implant and potential sources of error during placement. Furthermore, to obtain a more objective assessment of instability in the stifle joint in the future, the drawer test should be measured in millimeters and performed by multiple examiners.

## Data Availability

The raw data supporting the conclusions of this article will be made available by the authors, without undue reservation.
